# Michael James (1940–2023)

**DOI:** 10.1107/S2059798323006976

**Published:** 2023-09-15

**Authors:** J. N. Mark Glover, Cyril M. Kay, Joanne Lemieux, Randy J. Read

**Affiliations:** a Department of Biochemistry, Medical Sciences Building, University of Alberta, Edmonton, Alberta T6G 2H7, Canada; b Department of Haematology, Cambridge Institute for Medical Research, University of Cambridge, The Keith Peters Building, Hills Road, Cambridge CB2 0XY, United Kingdom

**Keywords:** obituaries, Michael James

## Abstract

Michael James is remembered.

Michael James ▸ liked to tell a story from his childhood when he was fascinated by rockets and their propellants. After walking over to the local branch of Canadian Liquid Air in Winnipeg he asked for some liquid oxygen. ‘Sure, kid’ they said, and put some in his bucket. (Attitudes to health and safety were different in those days.) As he walked home, he realized that liquid oxygen boils pretty quickly, so he ran the last part of the way and carried the rapidly emptying bucket into the basement. There was only a bit left and there wasn’t time to do anything fancy, so he splashed in some methanol to make a lox/alcohol propellant and tossed in a match. When he picked himself up from where he had been thrown by the explosion, he heard picture frames crashing down upstairs and his mother shouting ‘Michael, what have you done this time?’. He never lost this childhood enthusiasm for science (though his experiments became less dangerous) and was active in research until the end of his life.

Michael James was born in Vancouver and grew up in Winnipeg, Manitoba on the Canadian prairies. After high school, he studied chemistry at the local University of Manitoba, where he was excited to learn that it was possible to see the 3D structures of molecules using X-ray crystallography. Aiming to get the best possible background for a future working in this area, he applied to do a DPhil at Oxford with Dorothy Hodgkin in 1963. It was a time of great advances in crystallography, with Dorothy about to win the Nobel Prize for her ground-breaking structures of penicillin and vitamin B_12_, and the first protein structures having recently been determined in Cambridge. Michael’s thesis project was to determine structures of antibiotic peptides. While there, he was exposed to protein crystallography through the work going on at the same time to determine the 3D structure of insulin.

After completing his DPhil in 1966, Michael took a postdoctoral position determining small-molecule structures in the Department of Chemistry at the University of Alberta in Edmonton, Canada. He remained at the University of Alberta for the rest of his life. However, his career took a quick turn when he learned that the Biochemistry Department was seeking to set up a protein crystallography laboratory. With his move to Biochemistry he became the first protein crystallographer in Canada, determining the first 3D protein structure (that of *Streptomyces griseus* protease B, a serine protease) in 1974 (Delbaere *et al.*, 1975 ▸). That same year, Michael joined Larry Smillie, Cyril Kay, Brian Sykes, Robert Hodges and Robert Fletterick in forming the Medical Research Council (MRC) Group in Protein Structure and Function, later to be joined by Wayne Anderson, Charles Holmes and Zygmunt Derewenda. The Group became internationally known and respected for its multi-disciplinary approach to problems in protein chemistry as well as a superb environment for research training. During its tenure (1974–2000), the Group published over 1600 research papers and trained some 250 graduate students and postdoctoral fellows, many of whom went on to distinguished independent careers of their own.

In his research, Michael brought the same expectations of high precision to proteins that he had learned in small-molecule crystallography, and his group became known for the quality of the structures it produced, becoming early adopters of least-squares refinement (Sielecki *et al.*, 1979 ▸) and computer-graphics techniques. In the earlier years, Anita Sielecki (a member of the group since 1976 who eventually became his second wife) played a pivotal role in many aspects of the research including the development of optimal refinement protocols, as well as guiding students and creating a warm and supportive group atmosphere.

Initially, Michael concentrated his group’s efforts on understanding the structure and function of proteases, an area that remained important to him through the rest of his career. The group worked on a variety of serine proteases and their complexes with both small molecules (Brayer *et al.*, 1979 ▸) and protein inhibitors (Fujinaga *et al.*, 1982 ▸). They determined the first structure of an aspartyl protease, penicillopepsin (James *et al.*, 1977 ▸), setting the stage for the understanding of key members of this family such as renin (Sielecki *et al.*, 1989 ▸) and the HIV protease.

Eventually, the James laboratory turned its interest to the proteolytic enzymes from picornaviruses. Members of the laboratory determined the structures of the 3C proteinases from hepatitis A virus (Allaire *et al.*, 1994 ▸) and polio virus (Mosimann *et al.*, 1997 ▸), as well as the 2A proteinase from rhinovirus (Petersen *et al.*, 1999 ▸). All of these molecules are potential targets for antiviral drug development.

Prior to 2004, five families of proteolytic enzymes were known. A sixth family, the glutamic peptidases, was discovered in a collaboration between the James laboratory and Professor Kohei Oda of Kyoto, Japan (Fujinaga *et al.*, 2004 ▸).

Branching out into other areas, Michael’s group carried out structural studies of a variety of biochemically and medically important proteins. The structure of troponin-C (Herzberg & James, 1985 ▸) shed light on how muscle contraction is regulated. Structures of TEM-1 β-lactamase (Strynadka *et al.*, 1994 ▸) and its complex with the inhibitor protein BLIP (Strynadka *et al.*, 1996 ▸) gave new insight into antibiotic resistance and how it might be prevented. He also became interested in a structural understanding of inherited metabolic diseases, with a series of structures shedding light on Sandhoff (Mark *et al.*, 2003 ▸) and Tay–Sachs (Lemieux *et al.*, 2006 ▸) diseases, as well as mucopolysaccharidosis I (Bie *et al.*, 2013 ▸).

In collaboration with the Canadian company ViroChem Pharma, members of the James laboratory determined the structures of allosteric inhibitors of NS5B, the RNA-dependent RNA polymerase from hepatitis C virus (Wang *et al.*, 2003 ▸). The company took several of these non-nucleotide inhibitors to Phase II clinical trials.

His last published paper, on a protease from a virus that causes porcine epidemic diarrhoea, was special in having his daughter Michelle as a coauthor (Shamsi *et al.*, 2022 ▸).

Michael received many honours during his long and distinguished career. He was elected as a Fellow of the Royal Society of Canada in 1985 and as a Fellow of the Royal Society of London in 1989. He was presented the Martin Buerger Award of the American Crystallographic Association in 2009. In 2010, he received an honorary doctorate of science from his undergraduate alma mater, the University of Manitoba. In June of 2023, the month before he died, he was appointed an Officer of the Order of Canada, one of Canada’s highest honours.

In his approach to science, Michael’s passion was always to move closer to the truth. As a consequence, he took as much pleasure in the scientific advances of others in the MRC Group and the Department of Biochemistry as he did in his own group’s breakthroughs. He wanted to contribute and to understand, but was always unassuming; he could laugh at himself and never dominated or inflated his ego at the expense of others. Beloved by members of his laboratory, Michael was an inspirational friend and mentor to his students, postdoctoral associates and colleagues, and a joyous companion to those lucky enough to know him. ▸

Michael James died on the 24 July 2023 after a short admission to the University of Alberta Hospital. He is survived by his three children, Daphne and Marcus from his first marriage to Pat, and Michelle from his third marriage to Deborah.

## Figures and Tables

**Figure 1:**
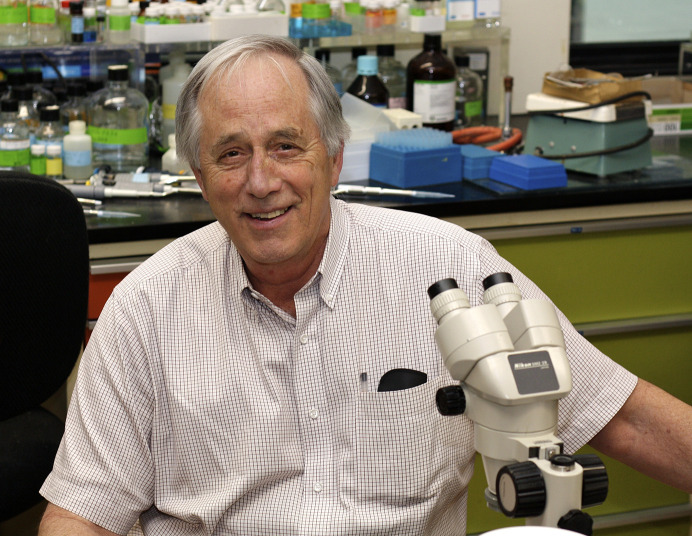
Michael at the laboratory bench in 2010.

**Figure 2:**
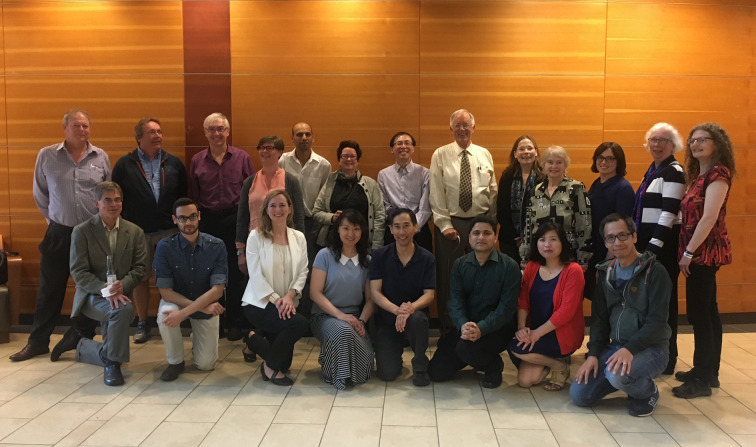
Michael with former laboratory members at a celebration of his career in June 2017, at the University of Alberta in Edmonton. From left to right, back row: Bret Church, Stanley Moore, Randy Read, Kathy Bateman, Amir Khan, Helen Blanchard, Ken Ng, Michael James, Natalie Strynadka, Maia Cherney, Nina Bernstein, Cathy McPhalen, Marie Fraser; front row: Steve Sprang, Rabih Farraj, Joanne Lemieux, Chunying Niu, Jiang Yin, Pravas Kumar Baral, Haiying Bie, Meitian Wang.
